# Serum Golgi protein 73 is not a suitable diagnostic marker for hepatocellular carcinoma

**DOI:** 10.18632/oncotarget.14954

**Published:** 2017-01-01

**Authors:** Tianhui Liu, Mingjie Yao, Shuhong Liu, Lu Wang, Leijie Wang, Jinlin Hou, Xiong Ma, Jidong Jia, Jingmin Zhao, Hui Zhuang, Fengmin Lu

**Affiliations:** ^1^ Department of Microbiology & Infectious Disease Center, School of Basic Medicine, Peking University Health Science Center, Beijing 100191, P.R. China; ^2^ Liver Research Center, Beijing Friendship Hospital, Capital Medical University, Beijing Key Laboratory of Translational Medicine in Liver Cirrhosis & National Clinical Research Center of Digestive Diseases, Beijing 100050, P.R. China; ^3^ Department of Pathology and Hepatology, Beijing 302 Hospital, Beijing 100039, P.R. China; ^4^ State Key Laboratory of Organ Failure Research, Guangdong Provincial Key Laboratory of Viral Hepatitis Research, Department of Infectious Diseases, Nanfang Hospital, Southern Medical University, Guangdong Province 510515, P.R. China; ^5^ State Key Laboratory for Oncogenes and Related Genes, Key Laboratory of Gastroenterology and Hepatology, Ministry of Health, Division of Gastroenterology and Hepatology, Renji Hospital, School of Medicine, Shanghai Jiao Tong University, Shanghai Cancer Institute, Shanghai Institute of Digestive Disease, Shanghai 200001, P.R. China

**Keywords:** Golgi protein 73, hepatocellular carcinoma, diagnostic marker, cirrhosis

## Abstract

Golgi protein 73 (GP73) has been suggested as a serum marker for the diagnosis of hepatocellular carcinoma (HCC). However, this has been challenged in recent years. In the present study, we found that the serum GP73 increased in HCC patients with cirrhosis but not in those without cirrhosis. The receiver operating characteristic curve (ROC) analysis demonstrated that serum GP73 had poor performance for differentiating HCC patients from cirrhosis patients. In addition, the immunohistochemistry revealed that aberrant expression of GP73 was primarily observed in cirrhotic and tumor liver tissues from both cirrhosis and HCC patients, but rarely in non-cirrhotic liver tissues from HCC patients without cirrhosis. Moreover, serum Alpha-fetoprotein in HCC patients with cirrhosis decreased sharply after resection of tumor tissue, while the serum GP73 remained stable. These data indicated that the background of cirrhosis was related to the elevation of serum GP73 in HCC patients. In conclusion, serum GP73 is not a suitable diagnostic marker for HCC.

## INTRODUCTION

Chronic liver diseases (CLD) can lead to liver fibrosis, cirrhosis and hepatocellular carcinoma (HCC). HCC represents more than 90% of primary liver cancers and is a major global health problem [1–4]. In order to improve patients’ prognosis and long-term survival, early diagnosis of HCC is essential to implement curative interventions [5]. Alpha-fetoprotein (AFP) is the most commonly used serological biomarker for HCC [6, 7]. However, the clinical diagnostic accuracy of AFP is unsatisfactory due to low sensitivity and specificity, and is no more recommended by European Association for the Study of the Liver (EASL) [8, 9].

Golgi protein 73 (GP73) is a resident Golgi transmembrane glycoprotein [10]. In normal liver, GP73 is primarily expressed in biliary epithelial cells but rarely in hepatocytes, while increased GP73 expression in hepatocytes appears in advanced liver disease regardless the etiology [11]. Recently, serum GP73 has been reported as a potential marker for diagnosing HCC [12–16]. However, some studies showed that serum levels of GP73 in HCC patients were markedly overlapped with [13, 17, 18] or even lower than those in cirrhotic patients [19, 20]. This may compromise its diagnostic accuracy because most HCC cases develop from cirrhosis [21–23]. Therefore, it is important to further evaluate the diagnostic value of serum GP73 for HCC.

In this retrospective study, the diagnostic performances of serum GP73 to differentiate HCC from pre-cirrhotic CLD or cirrhotic populations were evaluated. In addition, whether the background of cirrhosis accounts for the elevated serum GP73 in HCC patients were explored.

## RESULTS

### Clinical characteristics of patients

**Figure 1:**
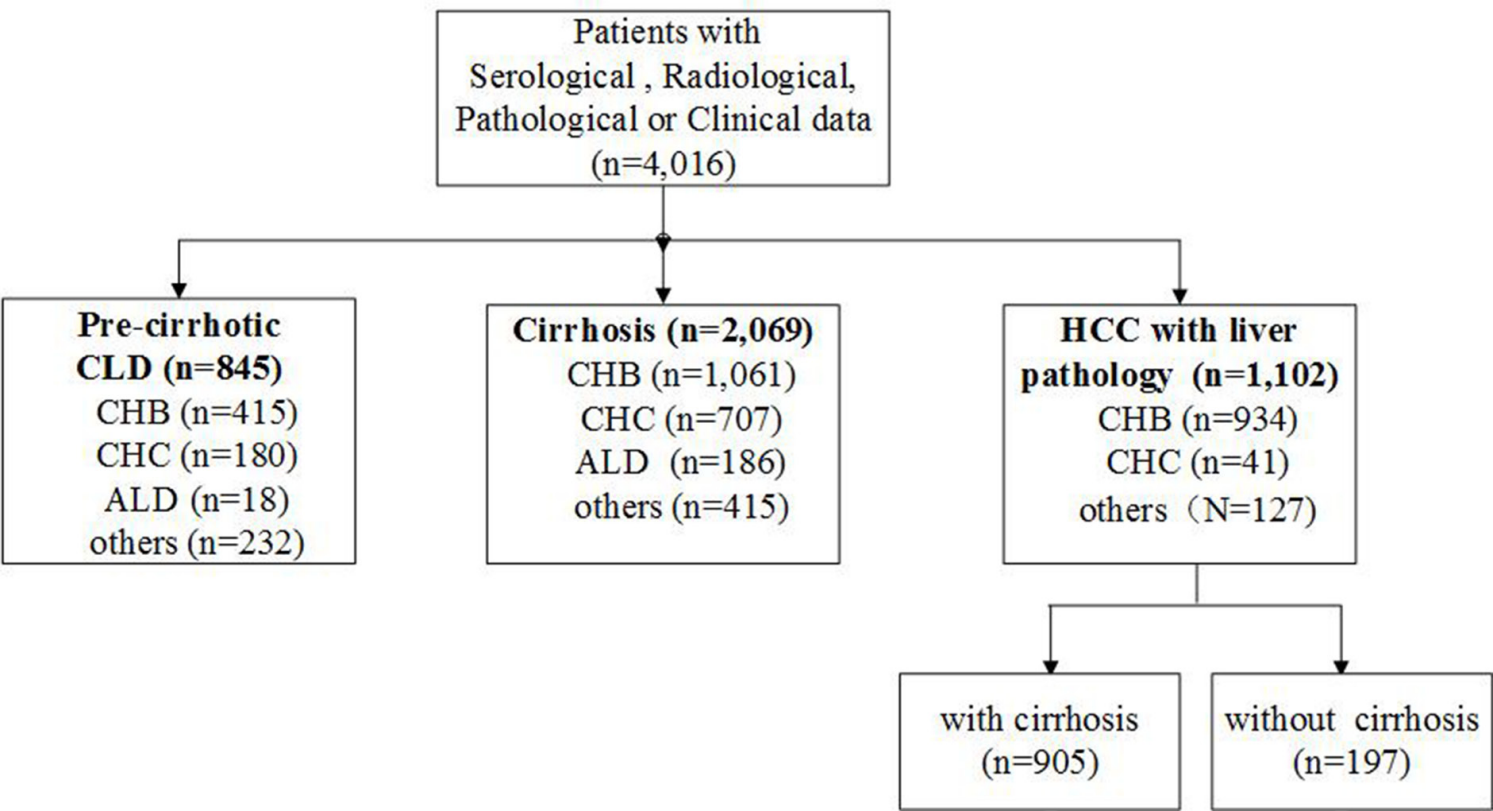
Patients’ flowchart, data provided in absolute numbers

From January 2010 to March 2016, 4,016 CLD patients in Beijing 302 Hospital who fulfilled the study criteria were enrolled. The flowchart of patients is shown in Figure 1 and patient’s characteristics are given in Table 1.

**Table 1:** Demographic and laboratory characteristics of 4,016 patients

Variables	Pre-cirrhotic CLD	Cirrhosis	HCC (*n* = 1102)	*P*
	(*n* = 845)	(*n* = 2069)		
Sex (Male/Female)	510/335	1283/786	938/164	0.000
Age (years)*	45.00 (37.00–52.00)	50.00 (44.00–58.00)	52.00 (45.00–59.00)	0.000
BMI(kg/m^2^)*	24.49 (22.03–26.59)	23.88 (21.64–26.42)	23.88 (21.71–25.95)	0.019
GP73 (ng/ml)*	43.60 (28.24–61.19)	133.70 (86.19–197.85)	100.40 (60.66–161.80)	0.000
AFP (ng/mL)*	2.13 (1.46–3.12)	3.17 (1.76–8.08)	34.1 (14.60–847.55)	0.000
ALT (U/L)*	26.00 (16.00–49.00)	30.00 (20.00–56.00)	36.00 (24.00–60.00)	0.000
AST (U/L)*	25.00 (19.00–37.00)	42.00 (28.00–72.00)	40.00 (28.00–70.00)	0.000
PLT (10^9^/L)*	182.50 (149.00–220.00)	90.00 (57.00–138.00)	135.00 (89.00–181.50)	0.000

Note: *Quantitative variables are expressed as median (P25, P75) for abnormal distribution.

Abbreviation: BMI = Body mass index, GP73 = Golgi protein 73, AFP = Alpha-fetoprotein, ALT = Alanine transaminase, AST = Aspartate transaminase, PLT = Platelet. *P* values were calculated by chi-square test or Kruskal-Wallis test. *P* value of < 0.05 (two sided) was considered as significant and written in bold text.

### Serum levels of GP73 increased significantly in both cirrhosis patients and HCC patients with cirrhosis, but not in HCC patients without cirrhosis

**Figure 2:**
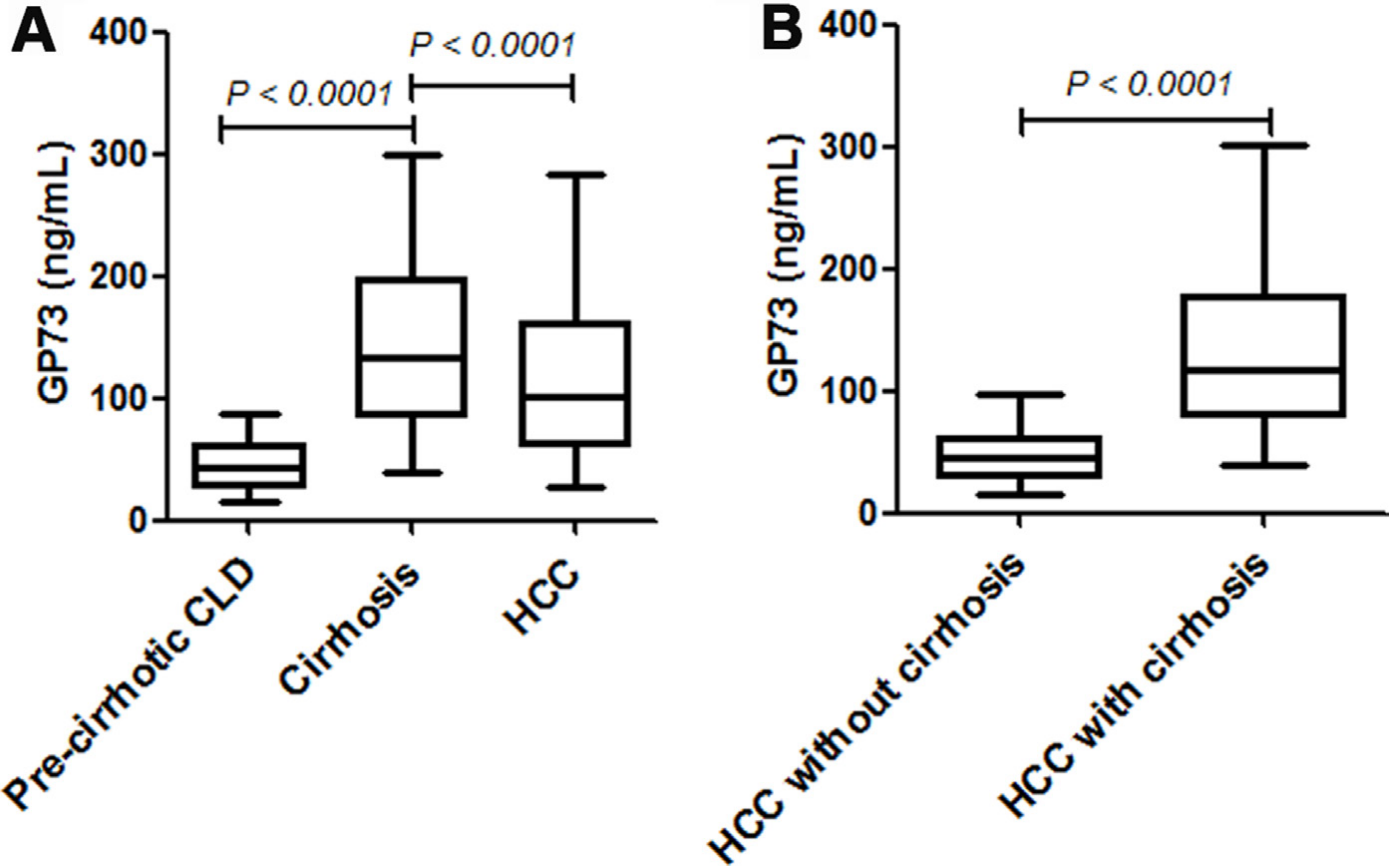
The serum levels of GP73 in different patient populations Data were represented as median (IQR). Significant differences were determined using Mann-Whitney *U* tests. (**A**) Serum levels of GP73 in pre-cirrhotic CLD, cirrhosis and HCC patients. (**B**) Serum levels of GP73 in HCC patients with and without cirrhosis.

Serum levels of GP73 in HCC patients were significantly higher than that in pre-cirrhotic CLD group (median [interquartile range (IQR)], 133.70 [86.19–197.85]) ng/ml *vs*. (median [IQR], 43.60 [28.24–61.19]) ng/ml, (*P* < 0.0001). However, a noticeable increase of serum GP73 was also observed in those HCC tumor-free cirrhosis patients, which was even higher than those of HCC patients (median [IQR], 100.40 [60.66 - 161.80]) ng/ml *vs*. (median [IQR], 133.70 [86.19 - 197.85]) ng/ml, (*P* < 0.0001) (Figure 2A).

As we know, most HCC cases developed from cirrhosis, to address whether the increase of serum levels of GP73 in HCC patients were cirrhosis related, the patients were then sub-grouped into the cirrhotic and free-cirrhotic HCC patient groups based on each patient’s cirrhotic background. The serum levels of GP73 were higher in HCC patients with cirrhosis, than in those without cirrhosis (median [IQR], 122.00 [79.58–180.50] ng/ml *vs*. 49.44 [32.19–67.09]) ng/ml, (*P* < 0.0001) (Figure 2B). Taken together, these results suggested that the elevated serum GP73 observed in HCC patients was possibly cirrhotic background related, but not HCC itself.

### Serum GP73 failed to distinguish HCC from cirrhotic patients

**Figure 3:**
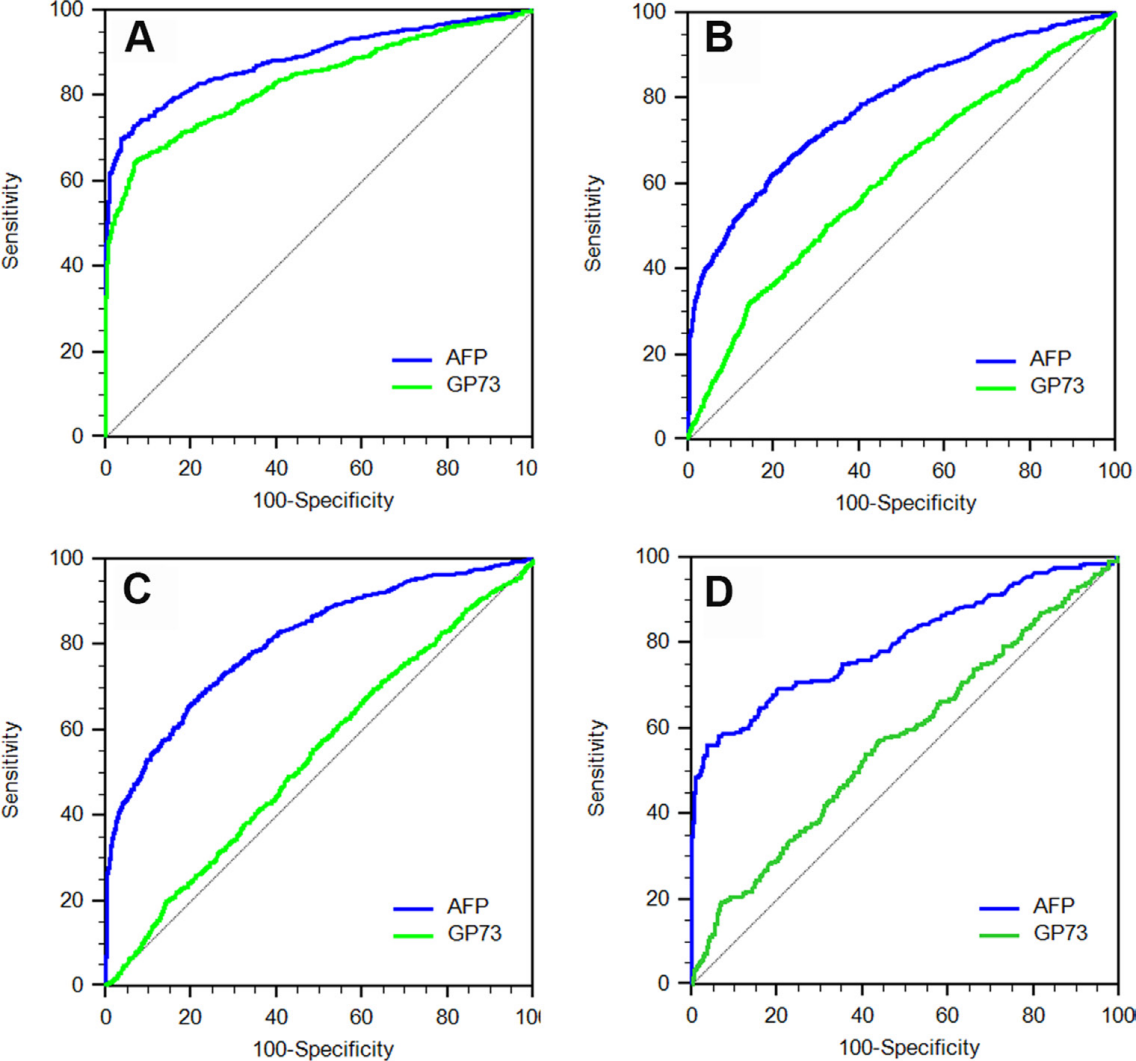
The receiver operating characteristic (ROC) curves of serum GP73 and AFP for diagnosis of HCC in different patient populations (**A**) ROC curve for differentiating HCC patients from pre-cirrhotic CLD patients. (**B**) ROC curve for differentiating HCC patients from cirrhosis patients. (**C**) ROC curve for differentiating HCC patients with cirrhosis from cirrhosis patients. (**D**) ROC curve for differentiating HCC patients without cirrhosis from pre-cirrhotic CLD patients.

The above results and previous reports [13, 17–17] indicated that the serum levels of GP73 in HCC patients were markedly overlapped with, or even worse, lower than that in cirrhotic patients. So it is reasonable to doubt the diagnostic value of serum GP73 for HCC. As shown in Figure 3A, though a 0.834 (95% CI: 0.816–0.850, *P* < 0.0001) area under the ROC curve for GP73 made it able to distinguish HCC patients from those pre-cirrhotic CLD patients, it dropped to 0.613 (95% CI: 0.595–0.630, *P* < 0.0001) when cirrhotic patients were used as non-HCC control (Figure 3B). Considering that most HCC patients were developed from cirrhosis, and serum GP73 could not accurately distinguish HCC patients from those cirrhotic patients free of HCC.

To avoid the possible effect of the background of cirrhosis, the diagnostic values of serum GP73 in HCC patients with cirrhosis and without cirrhosis were then analyzed respectively. The AUROC of GP73 was 0.527 (95% CI: 0.542–0.601) to distinguish HCC patients with cirrhosis from cirrhosis patients, and was 0.538 (95% CI: 0.520–0.557) when to distinguish HCC patients without cirrhosis from pre-cirrhotic CLD patients, respectively (Figure 3C, 3D). In contrast, AFP remained a good diagnostic marker for HCC patients regardless the background of cirrhosis.

### Serum levels of GP73 in HCC patients remained stable after tumor tissue resection

**Figure 4:**
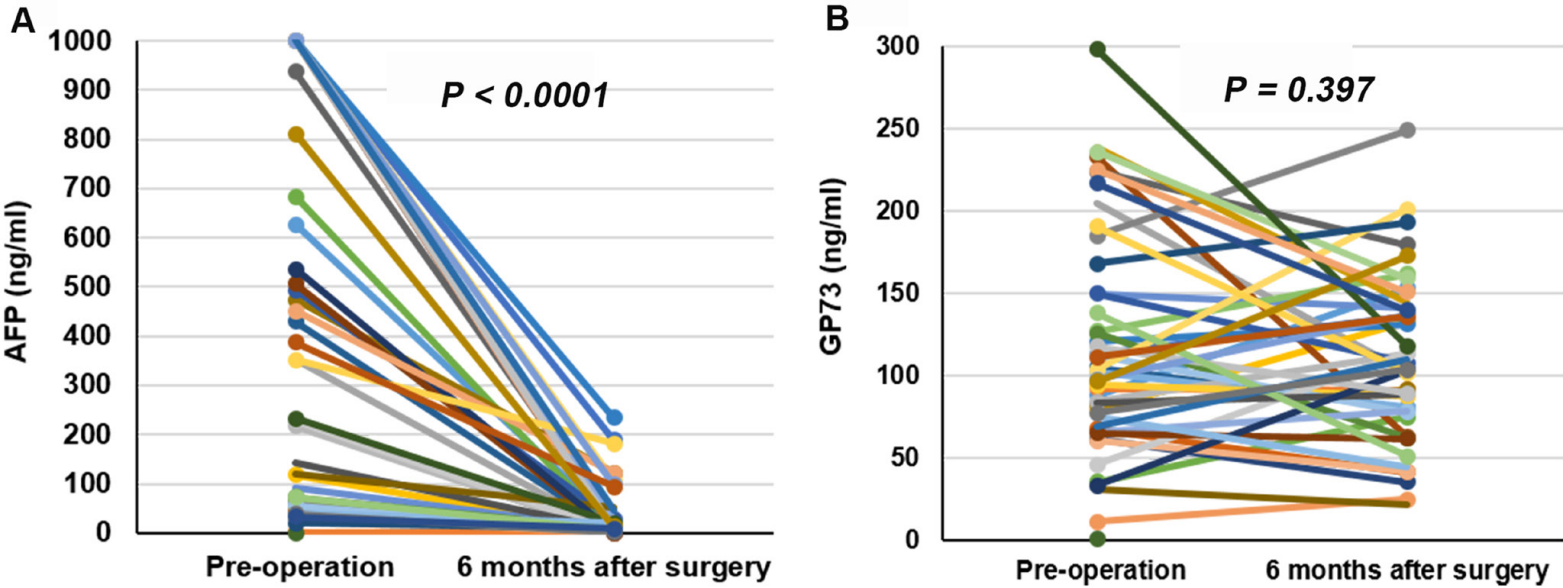
Dynamic changes of serum AFP and GP73 after operation (**A**) Dynamic changes of serum AFP after operation. (**B**) Dynamic changes of serum GP73 after operation.

To provide further evidence that the elevated serum GP73 in HCC patients was not tumor related, the dynamic change of pre- and post-operation serum AFP and GP73 were observed in a small group of HCC patients who had underwent curative resection. All of the 113 HCC patients in this subgroup had no tumor reoccurrence within half a year after curative operation. The serum levels of AFP in HCC patients decreased dramatically after tumor tissue resection (median [IQR], 246.00 [62.52–845.80] ng/ml *vs*. 5.24 [2.14–20.10]) ng/ml (*P* < 0.0001), while the serum levels of GP73 remained stable (median [IQR], 96.53 [61.47–150.20] ng/ml *vs*. 93.38 [61.61–136.70]) ng/ml (*P* = 0.397) (Figure 4).

### GP73 predominantly expressed in cirrhotic tissues regardless of HCC

**Figure 5:**
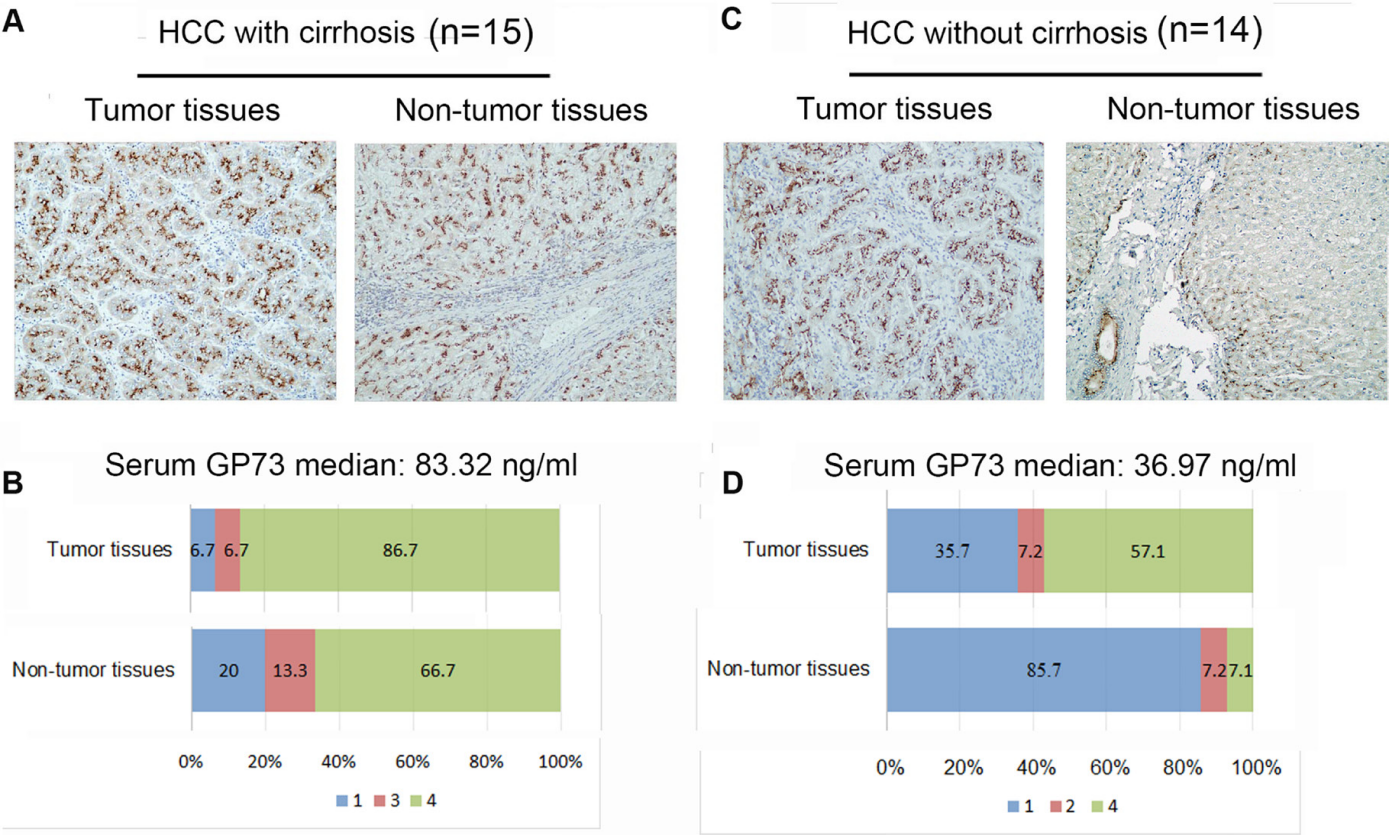
Immunoreactivity of GP73 in liver tissues from HCC patients (**A** and **B**) Representative immunoreactivity of GP73 in tumor and cirrhotic tissues from the same HCC patient with cirrhosis (*n* = 15). (**C** and **D**) Representative immunoreactivity of GP73 protein in tumor and non-tumor liver tissues from the same HCC patient without cirrhosis (*n* = 14). According to the average percentage of GP73 positive hepatocytes in ten high power fields (×400) of each sample, the immunoreactivity of GP73 was graded as 0–5% (0), 6%–25% (1), 26%–50% (2), 51–75% (3), 76%–100% (4).

The results above suggested that the elevated serum GP73 was not HCC tumor but cirrhotic background related. To further confirm this speculation, the expression of GP73 in the liver tissue derived from a small group of HCC patients was visualized by immunohistochemistry. As shown in Figure 5A and 5B, large amount of GP73 positive staining cells could be seen in both tumor and non-tumor liver tissues from cirrhotic HCC patients. In detail, strong immunoreactivity (score 4) were seen both in tumor tissues (14/15, 93.3%) and in paired non-tumor tissues (12/15, 80.0%) from HCC patients with cirrhosis. In those HCC patients without cirrhosis, strong immunoreactivity of GP73 were seen in tumor tissue (8/14, 57.1%), but seldom in paired non-tumor liver tissues (1/14, 7.1%) (Figure 5C and 5D).

Moreover, the patterns of GP73 expression in tumor cells and in hepatocytes within the non-tumor nodules are greatly different. In tumor tissues from HCC patients, GP73 showed a pattern with diffuse coarse-block pattern in perinuclear that concentrated near the lumen of glandular structures, or inside peri-cytomembrane between two layers of hepatocytes. However, in paired cirrhotic non-tumor tissues from the HCC patients, as well as from the CLD patients with cirrhosis, diffuse fine-granular in the cytoplasm was the major pattern of GP73 distribution.

The following multivariate analysis provided additional evidence not to support GP73 as a HCC diagnostic marker. There were no correlation between serum GP73 levels and the biological characteristics of HCC, including tumor size, degree of tumor differentiation, tumor-node-metastasis (TNM) stage and AFP. In contrast, the results indicated that higher serum GP73 levels were independently associated with several fibrosis/cirrhosis relevant parameters, such as higher gamma glutamyl transpeptidase, total bile acid, PT and lower albumin, respectively (Table 2).

**Table 2:** Predictive variables for increased GP73 by multivariate analysis using linear regression analysis model in patients with HCC

Variables	Unstandardized Coefficients		Standardized Coefficients	*P*	95.0% CI	
	B	Std. Error	Beta		Lower Bound	Upper Bound
(Constant)	30.87	100.86		0.760	–167.46	229.20
Age	0.21	0.27	0.03	0.435	–0.32	0.75
Height	0.39	0.53	0.04	0.466	–0.66	1.43
Weight	–0.17	0.26	–0.03	0.503	–0.69	0.34
Tumor size	0.78	0.93	0.04	0.399	–1.04	2.61
Tumor differentiation degree	–1.24	6.43	–0.01	0.848	–13.88	11.41
TNM stage	3.72	3.36	0.05	0.269	–2.89	10.33
PLT	0.03	0.05	0.03	0.575	–0.07	0.13
ALB	–2.40	0.71	–0.18	0.001	–3.79	–1.00
PA	–0.13	0.06	–0.12	0.034	–0.26	–0.01
TBiL	–0.03	0.07	–0.02	0.705	–0.16	0.11
ALT	0.00	0.04	0.02	0.898	–0.07	0.08
AST	–0.03	0.04	–0.08	0.519	–0.11	0.05
ALP	–0.02	0.04	–0.03	0.499	–0.10	0.05
GGT	0.12	0.03	0.20	0.000	0.07	0.18
TBA	0.27	0.13	0.11	0.044	0.01	0.53
CHE	0.00	0.00	0.00	0.927	0.00	0.00
CEA	0.00	0.00	–0.03	0.513	0.00	0.00
AFP	0.00	0.00	–0.04	0.307	–0.01	0.00
PT	6.72	1.94	0.16	0.001	2.90	10.54
INR	–4.22	4.88	–0.04	0.387	–13.81	5.37

* Abbreviation: PLT = Platelet, ALB = Albumin, PA = Prealbumin, Tbil = Total bilirubin, ALT = Alanine transaminase, AST = Aspartate transaminase, ALP = Alkaline phosphatase, GGT = Gamma glutamyl transpeptidase, TBA = Total bile acid, CHE = Cholinesterase, CEA = Carcino-embryonic antigen, AFP = Alpha-fetoprotein, PT = Prothrombin time, INR = International normalized ratio.

## DISCUSSION

In the present study, we found that serum GP73 increased only in HCC patients with cirrhosis, but not in those without cirrhosis. The AUROC of serum GP73 for diagnosing HCC was as low as 0.613 (95% CI: 0.595–0.630, *P* < 0.0001) when cirrhotic patients were used as controls. In addition, our data also showed that serum GP73 could not distinguish HCC patients with cirrhosis from cirrhosis patients. These data strongly suggested that serum GP73 has no diagnostic value for HCC.

Different from the report by Mao *et al*. who claimed that serum GP73 has high sensitivity and specificity in the diagnosis of HCC [12], the results here clearly excluded that serum GP73 is a good diagnostic marker for HCC. The main reason for this discrepancy could be the selection of non-HCC control groups. In Mao’s study, the control group composed more healthy subjects and HBV carriers but less cirrhotic patients, and this precluded them evaluating the performance of serum GP73 to differentiate HCC from cirrhosis. Considering that most HCC cases develop from cirrhosis [21–23], their conclusion is of limited significance or even misleading in real clinical scenario. Though some other studies thereafter did include cirrhosis in the control group, the relatively small numbers of patients prevented them from reaching convincing conclusion [13, 14].

Different with the dramatic decrease of serum AFP in HCC patients with cirrhosis, the serum levels of GP73 remained stable resection of tumor tissue. In addition, we found that serum levels of GP73 in patients with HCC were not correlated with tumor size and differentiation status.

Serum levels of GP73 were highly correlated with the *in situ* GP73 expression in non-tumor liver tissues from HCC patients, no matter whether there is cirrhotic background or not. Furthermore, immunohistochemistry demonstrated that increased expression of GP73 was only observed in cirrhotic liver tissues of patients regardless of HCC. Interestingly, the expression of GP73 showed different pattern in tumor cells and in aberrant regenerative hepatocytes in nodules. GP73 mainly localized around nuclear in tumor cells, while mainly localized in the cytoplasm in cirrhotic nodules. These data indicated that GP73 might have different biological functions in tumor cells and in aberrant regenerative hepatocytes in nodules. However, further studies are needed to explore the possible pathological role both in HCC and cirrhosis.

Put together, all these evidences suggested that it is the cirrhotic background of the liver but not the HCC itself that is associated with the elevation of serum GP73 in HCC patients. Consistent with our findings, Qiao *et al*. had also noticed that the increased serum GP73 in CHB patients with cirrhosis [24], and GP73 positive cells in the liver were gradually increased with the severity of liver fibrosis [25]. Therefore, serum GP73 could be considered a potential marker for cirrhosis. Of note, further studies are needed to evaluate the diagnostic performance of serum GP73.

In conclusion, we demonstrated that the cirrhotic background of the liver is associated with the elevation of serum GP73 in HCC patients, and serum GP73 is not a marker for HCC diagnosis.

## MATERIALS AND METHODS

### Patients

This retrospective study recruited consecutive CLD patients between January 2010 and March 2016 in Beijing 302 Hospital with pre-cirrhotic CLD, cirrhosis as well as HCC (Table 1). The enrolled HCC patients either underwent percutaneous liver biopsy or curative surgery had been diagnosed by pathologic examination following the Practice Guidelines [26].

For CLD patients, the diagnosis of chronic hepatitis B (CHB) was based on hepatitis B surface antigen (HBsAg) positive for more than 6 months, with clinical or laboratory signs of chronic hepatitis [29]. The diagnosis of chronic hepatitis C (CHC) was based on the detection of both hepatitis C virus (HCV) antibodies and HCV RNA in the presence of signs of chronic hepatitis [30]. The diagnosis of alcoholic liver disease (ALD) was based on documentation of excess alcohol consumption (> 30 g/d) and the presence of clinical and/or biological abnormalities suggestive of liver injury [31]. The other CLD patients, including non-alcoholic fatty liver disease (NAFLD), autoimmune hepatitis (AIH) and primary biliary cirrhosis (PBC), were diagnosed per corresponding guidelines [32–34].

For the clinically diagnosis of cirrhosis, one of the following criteria should be met: 1. Endoscopy: esophageal varices, exclusion of non-cirrhotic portal hypertension. 2. If no endoscopy, two of the following criteria should be met: 2.1 Typical findings of CT or MRI with one of the following observations: irregular liver surface, granular or nodular liver parenchyma, with or without splenomegaly (thickness of spleen > 4 cm or > 5 pedicle-rib units). 2.2 Platelet count of less than 100,000/mm3 excluding the other possible causes. 2.3 Serum albumin less than 3.5 g/dL, or prothrombin time (PT) prolonged or international normalized ratios (INR) > 1.3 (anticoagulants or thrombolytic drugs discontinued more than 7 days) [27, 28]. For pre-cirrhotic CLD cases, the CLD patients who fulfilled the above criteria for clinically diagnosis of cirrhosis were excluded.

This study was approved by the Ethics Committee of Beijing 302 Hospital and informed consent forms were signed by the participants.

### Measurement of serum levels of GP73 and AFP

Quantitative detection of serum GP73 was performed by using commercially available double-antibody sandwich enzyme-linked immunosorbent assay (ELISA) kit (Hotgen Biotech Inc., Beijing, China), according to the manufacturer’s protocol. Serum AFP was determined by using electrochemiluminescence immunoassay system Cobas E601 (Roche, Mannheim, Germany).

### Immunohistochemistry

Deparaffinized sections from tissue were microwaved in 10 mM citrate buffer (pH 6.0), exposed to 3% hydrogen peroxide for 20 min and blocked with 25% goat serum for 45 min. The sections were incubated with rabbit anti-GOLPH2 antibodies (ab109628, 1:1000 dilution; abcam, Cambridge, UK) for 2 h at 37°C incubator, and then incubated with Universal anti-Mouse/Rabbit-HRP (D-3004, Supervision) for 30min at room temperature. The staining of GP73 was visualized using DAB color kit (MXB).

According to the average percentage of GP73 positive hepatocytes in ten high power fields (×400) of each sample, the immunoreactivity of GP73 was graded as 0–5% (0), 6%–25% (1), 26%–50% (2), 51–75% (3), 76%–100% (4).

### Statistical analysis

All statistical analyses were performed with MedCalc (15.8.1) software. The difference between groups was tested using the Kruskal-Wallis test. The area under the receiver operating characteristic (ROC) curve (AUC) was used to evaluate the diagnostic performance. All tests of significance were two-tailed and *P* < 0.05 was considered statistically significant.
